# Prioritization of Medical Errors in Patient Safety Management: Framework Using Interval-Valued Intuitionistic Fuzzy Sets

**DOI:** 10.3390/healthcare8030265

**Published:** 2020-08-12

**Authors:** Zeynep Tugce Kalender, Hakan Tozan, Ozalp Vayvay

**Affiliations:** 1 Department of Industrial Engineering, Faculty of Engineering, Marmara University, 34722 Istanbul, Turkey; tugce.simsit@marmara.edu.tr; 2 School of Engineering and Natural Sciences, Istanbul Medipol University, 34810 Istanbul, Turkey; 3 Faculty of Business, Marmara University, 34722 Istanbul, Turkey; ozalp@marmara.edu.tr

**Keywords:** decision-making, interval-valued intuitionistic fuzzy sets, medical errors, patient safety, quality management

## Abstract

Medical errors negatively affect patients, healthcare professionals, and healthcare establishments. Therefore, all healthcare service members should be attentive to medical errors. Research has revealed that most medical errors are caused by the system, rather than individuals. In this context, guaranteeing patient safety and preventing medical faults appear to be basic elements of quality in healthcare services. Healthcare institutions can create internal regulations and follow-up plans for patient safety. While this is beneficial for the dissemination of patient safety culture, it poses difficulties in terms of auditing. On the other hand, the lack of a standard patient safety management system, has led to great variation in the quality of the provided service among hospitals. Therefore, this study aims to create an index system to create a standard system for patient safety by classifying medical errors. Due to the complex nature of healthcare and its processes, interval-valued intuitionistic fuzzy logic is used in the proposed index system. Medical errors are prioritized, based on the index scores that are generated by the proposed model. Because of this systematic study, not only can the awareness of patient safety perception be increased in health institutions, but their present situation can also be displayed, on the basis of each indicator. It is expected that the outcomes of this study will motivate institutions to identify and prioritize their future improvements in the patient safety context.

## 1. Introduction

Providing better hospital service quality is one of the most important issues in the modern healthcare industry [1]. The importance of healthcare services has been emphasized globally [2,3], where quality has been indicated as key to surviving in an extremely competitive environment [3]. At the end of the 20th century, many reports revealed that there must be a change to achieve high-quality levels of care while providing strong proof of evidence [4,5,6,7]. These reports have highlighted quality deficiencies as never before and explained the need for change in terms of quality.

At present, one of the most important topics in healthcare quality management is patient safety and rights [8]. Patient safety practices are listed to provide safe services to patients and health employees in all health institutions, with the main aim of regulating procedures and principles, thus increasing the quality level in health systems by determining the possible risks for patients and employees. In 2000, The Institute of Medicine (IOM) in the USA published a report called “To Err is Human: Building a Safer Health System” which has been considered a milestone in patient safety [5]. The report focused on patient safety, estimating that between 44,000 and 98,000 deaths occur annually in hospitals due to medical errors. Over half of these errors were determined to be avoidable; this would result not only in saved lives, but also in an estimated financial savings of $17–$29 billion per year. Over the decade after the IOM report was released, many efforts were made to reduce medical errors [9]; however, they are still the 14th leading cause of the global disease burden [10] and the 3rd biggest cause of death in the USA [11]. In the third Global Patient Safety Challenge [12], it was stated that awareness of patient safety is increasing globally day by day, revealing that the common point of applied and ongoing projects is the coordinated work of different units. At present, global understanding of safety and quality issues have improved, and several actions have been developed and implemented nationally [13,14,15]; furthermore, these developed frameworks and analyses have been shared internationally [10].

Maximizing patient safety is a fundamental case for every health institution; however, there is also a financial impact, as medical errors cause the additional use of healthcare resources, such as more diagnostic testing, longer hospitalization, and so on. According to an Organisation for Economic Co-operation and Development (OECD) report [16], almost 15% of hospital spending in OECD countries is used for treating safety failures. Considering the worldwide health spending of $7.6 trillion [17], prevention of medical errors provides an opportunity to minimize costs. It is a well-known fact that a considerable proportion of medical errors can be prevented with simple actions and patient safety protocols. Moreover, the preventability of medical errors has changed over time, due to the rapid increases in medical knowledge and technology. In such cases, a standard patient safety system in which medical errors can be prioritized may ensure the reliability of the measurement system before determining regulatory and preventive actions.

### 1.1. Definition of Medical Error

Medical error is an unintended physical injury resulting from or contributed to by medical care which requires additional monitoring, treatment, hospitalization, or that may even result in death. The determination of medical errors at the right time and revealing their core reasons are vital parts of their solution.

IOM [5] defined a medical error as: (i) the failure of a planned action to be completed as intended (error of execution) or (ii) the use of a wrong plan to achieve an aim (error of planning). The National Patient Safety Foundation in the USA [18] classified errors into three subcategories: (i) errors of commission (doing the wrong thing), (ii) errors of omission (not doing the right thing), and (iii) errors of execution (doing the right thing incorrectly). In the report called “Free from Harm” [19], an error was defined as: (i) an act of commission (doing something wrong) or (ii) omission (failing to do the right thing), which leads to an undesirable outcome or significant potential for such an outcome.

The safety reporting system generated by the Ministry of Health of Turkey is a platform, which health institutions and professionals can use to report medical errors, and, so, they can be informed of common errors and the precautions of these errors. Medical errors are classified into four main categories: (i) laboratory, (ii) medication, (iii) general/surgical, and (iv) patient-centered errors. In each category, there are also several sub-categories based on the procedure of the related system.

In healthcare systems, many factors influence the rate of errors. The possible reasons for errors are usually associated with some combination of the team, the task performed, and the patient. However, less-visible factors such as context, management, and work environment must also be addressed. There may be several reasons affecting and causing problems in patient safety, such as missed information, missed co-ordination, lack of control processes, non-standardized medical equipment, and so on. Recent studies have revealed that most medical errors are caused by the system rather than individuals. According to the study conducted by Blendon et al. [20], 35% of doctors and 42% of the general public believed that they or one of their family members had experienced a medical error during the treatment process. Schoen et al. [21] stated that 34% of the general public in the USA believed that they had experienced a medical error during the treatment process. King et al. [22] stated that the core reasons for the patient safety problem are lack of communication (70%), lack of training (60%), lack of patient evaluation (40%), and lack of labor (25%). Carayon et al. [23] stated that most errors and inefficiencies in patient care arise not from the solitary actions of individuals, but from the conflicting, incomplete, or sub-optimal systems of which they are a part and with which they interact.

To provide well-qualified healthcare services, medical establishments should meet basic requirements, should apply the process of patient safety perfectly in a flawless way, and should perform processes with the appropriate procedure. Health institutions are generally held responsible for the establishment of safety reporting systems by governments [24], in which medication safety, transfusion safety, surgical safety, patient falls, and so on are included. Corrective and preventive actions should be taken for reported events [25]. For that reason, emphasis should be placed on the system of care, not only to prevent errors but also to learn from error and build a culture of safety involving healthcare professionals, organizations, and patients.

### 1.2. Current State of the System in Turkey

In Turkey, quality improvement studies were started in 2003, in the context of the Health Transformation Program, following which standardization programs for hospitals have been developed and pilot studies with several trainings have been planned. More recently, quality indicators for healthcare have been determined within the scope of quality improvement studies. Additionally, a “classification system for patient safety errors” has been created by determining the error codes with the safety reporting system and, thus, the following-up of errors was targeted.

In the category of the laboratory-related errors, the errors can be separated into three sub-categories, taking into account the laboratory processes; these errors can occur during pre-analytic (PR), analytic (AN), and post-analytic (PO) processes. According to the report published by the Ministry of Health [26], 234,746 errors in the pre-analytic category, 24,886 analytic errors, and 8039 post-analytic errors have been reported. After the reported errors were examined, it was observed that “L18—Clotted sample”, “L17—Sample with hemolysis”, “L16—Insufficient sample”, “L05—Wrong Record”, and “L01—Wrong test request” were the top five medical errors. The report also focused on the time interval and the department that the error occurred in. The analysis revealed that most laboratory-related errors occur in the policlinic and emergency department during the 08.00–12.00 time interval. In general, a total of 74,383 error reports were made to the safety reporting system in 2016.

The second main category in the safety reporting system includes medication-related errors, which has been mostly reviewed as a drug safety issue in the literature. According to the safety reporting system of the Ministry of Health [26], for medication-related errors, there are six sub-categories representing all the steps of medication safety: storage (I0), demand (I1), preparation (I2), transfer (I3), implementation (I4), and post-implementation (I5). According to the published report (2016), 1119 storage errors, 5132 demand errors, 2307 preparation errors, 997 transfer errors, 1227 implementation errors, and 379 post-implementation errors were reported. The top five errors were “I1g—erroneous dosing”, “I1a—wrong drug demand”, “I0a—inappropriate temperature and humidity”, “I2b—preparation of the wrong drug”, and “I3c—transferring wrong drug from pharmacy”. Detailed analysis of the error reports revealed that medication-related errors generally occur in the 08.00–12.00 time interval and are mostly seen in clinics (66.52%) and pharmacies (18.43%). When the results of the reported errors were examined, another remarkable point was revealed: although there exists a prevention system in the current healthcare system, the errors “I1b—wrong drug selection in the electronic system”, “I1f—illegible handwriting”, and “I1i—unwritten verbal demand” were still reported.

The third main category, general/surgical errors, includes eight sub-categories: (i) clinical surgical procedure preparation (C1), (ii) transfer to the operating room and patient acceptance (C2), (iii) operating room surgical procedure (C3), (iv) pre-anesthesia preparation and control (C4), (v) controls before surgery (C5), (vi) follow-up and control during operation (C6), (vii) controls at the end of the operation (C7), and (viii) departure of the patient from the operation room and transfer (C8). According to the report (2016), 680 errors for C1, 135 errors for C2, 61 errors for C3, 107 errors for C4, 61 errors for C5, 19 errors for C6, 62 errors for C7, and 32 errors for C8 were reported. The top five errors were “di—non-marking of operation area/side”, “kd—unverified patient ID, location of operation and surgical procedure”, “gd—not confirming removal of make-up, prosthesis, and valuable items”, “tr—the operation area has not been shaved”, and “ts—health worker does not accompany patient transfer”.

The final main category includes patient-related errors, which has eight sub-categories: (i) physical structure-related (BYH), (ii) device/equipment/system-related (CSH), (iii) medical record and clinical evaluation-related (TKH), (iv) communication error (ILH), (v) care, diagnosis, and treatment process-related (BTH), (vi) patient/attendant-related (HRH), (vii) errors concerning the transfusion of blood and blood products (KTH), and (viii) errors regarding nutrition (NIH). According to the report [26], 156 physical structure-related errors, care, 286 diagnosis and treatment process-related errors, 271 patient/attendant-related errors, 73 communication-related errors, 138 devices/equipment/system-related errors, and 189 medical record and clinic evaluation-related errors were reported. The top errors were “HRHc—patient falls”, “TKHg—incorrect record of patient basic information”, and “TKHa—incorrect identification of patients”.

Turkish regulations emphasize the importance of patient safety [27], in which it is stated that health institutions (within the scope of regulation) are obliged to carry out their internal regulations and measures, in order to provide the necessary activities to ensure patient and employee safety (Ministry of Health Turkey, 2011). In simple terms, each health institution can determine its methods to provide patient safety; however, there exists no standard in this regard, even if they use already determined health indicators.

### 1.3. Aim of the Study

Healthcare institutions can create internal regulations and follow-up plans for patient safety. While this is beneficial for the dissemination of patient safety cultures, it poses difficulties in terms of auditing. The lack of a standard patient safety system for the evaluation of medical errors leads to great variation in terms of provided service quality levels among hospitals. Therefore, it is clear that there is a need for an effective, systematic, and standardized system. In this regard, this study aims to create an index system that provides a standard for the management of patient safety by prioritizing medical errors. However, the management of the safety system in healthcare institutions is a complex problem that requires hybrid models in which comprehensive analyses are to be made. Moreover, analyzing the system is quite difficult, as the healthcare system itself, and each of the factors affecting the system, are probabilistic and uncertain.

Fuzzy logic is one of the best methods in logical model processes to help people to make rational decisions in environments affected by uncertainty. After the development of fuzzy logic theory by Zadeh in 1965 [28], many applications in different directions have been made and, over time, the scope of the theory has been expanded to analyze different data systems. Intuitionistic fuzzy sets, which can be considered as an extended version of the classical fuzzy set theory, have been applied in such different fields as medical diagnosis [29,30], decision-making problems [31,32,33], and pattern recognition [34,35,36]. In intuitionistic fuzzy set theory, each choice is basically represented as a single point within the set. On the other hand, the data may exist in a structure that cannot be expressed in a single point, due to uncertainty or complexity, and should be regarded as a range, rather than a single point. In such cases, the use of interval-valued intuitionistic fuzzy sets (IVIFs) is preferred [37,38,39]. In a patient safety management, imperfect knowledge situations are inevitable and the structure of medical error data causes uncertainty in the decision-making process. For that reason, in this study, IVIFs are used in the analysis, not only due to the complexity of the data but also due to the need for the decision maker’s level of hesitation to be added into the analyses.

The rest of the paper is structured as follows: In the initial part of the study, a literature review considering patient safety assessment frameworks is carried out. Afterward, the preliminaries of each methodology, which is used in the proposed hybrid model, are explained, in detail, in order to create a better understanding. In the third part, the main problem is defined, and the current situation is given. Following this, the proposed model is explained step-by-step by presenting the results of the implementation. In the conclusion, limitations and future research directions are presented.

## 2. Patient Safety Management Methodologies

In general, patient safety-related problems are addressed by focusing on a single incident at a time; however, the core reasons are often not revealed, such that the same error may occur again [40]. Furthermore, in most cases, the core problems for patient safety are not related to the prevention or improvement mechanisms.

In 2007, the patient safety toolbox was published, which is one of the comprehensive studies of the SIMPATIE (Safety Improvements for Patients in Europe) project [41]. The project was designed to establish a systematic knowledge repository on patient safety and to define recommendations for the improvement of health services. In the report, some major patient safety methodologies were presented, as follows:

- Move Your Dot: In 2003, the Institute of Healthcare Improvement (IHI) in the USA [42] generated this model for safety management in hospitals, in which mortality rates are measured and evaluated, with the aim of reducing them. In this method, mortality rates, which are standardized based on several variables, are used to improve care by comparing hospitals with each other and the national average. After standardized mortality rates are calculated, mortality rates in determined hospitals can be measured and improvements can be planned to reduce them.
- Trigger tool: This tool is generally used to screen the current state and to identify adverse events [43,44]. Further analysis of the adverse events provides a starting point for improvements.
- Bow-tie model: A systematic model is generated, in which risks, preventive barriers, and recovery barriers of processes are detailed as a whole picture [45]. In the model, risk factors, preventive barriers, and recovery barriers are integrated. The route of an incident can be tracked, from the beginning to the consequences, by presenting the failures in preventive and recovery barriers [46].
- Health failure mode and effect analysis (H), FMEA: FMEA is a simple and widely used methodology, which basically prioritizes risks based on a calculated risk number. The main reason for the method’s extensive use is the simple structure of the method, which is focused on preventing deficiencies and improving safety [47,48].
- Root cause analysis (RCA): This method is generally used to evaluate an incident using two basic questions: “what happened?” and “why did it happen?” The main aim is to prevent the occurrence of similar accidents in the future by learning from the experience [49].
- Preventive and Recovery Information System for Monitoring and Analysis (PRISMA): The model is focused on processes, rather than individuals. A casual tree, which is a visual presentation of the related error and all of the information about it, is used as a starting point to classify basic causes. Then, the findings are translated into preventive measures [50].

These methodologies are examined in terms of their advantages/disadvantages and briefly explained in Table 1.

In addition to these above-mentioned commonly used models, there are several models that can be used within hybrid methodologies for safety management. Some of these methodologies are briefly explained, as follows:

- Benchmarking can be used to measure and compare the quality and safety of care. After the indicators are determined, the healthcare institution can make an assessment of its processes and take action accordingly. However, the main problem in benchmarking is the comparison of different populations. In these cases, external accountability is dangerous [51]. La Fata et al. [52] used benchmarking to compare the service performance of hospitals under a fuzzy evaluation environment and generated a hybrid decision-making model.
- Bundles, which is defined as a set of precautions, which are used collectively. Smorenburg [53] stated that caregivers generally tend to use simple bundles of care processes to make changes. The application of the bundle will be a successful and effective method in reaching a set of objectives. However, a limited number of interventions can be used to create bundles.
- Rapid response teams are used to improve communication between several care providers as supporting functions. Studies show that rapid response teams can be called if a nurse or doctor suspects that a patient is at risk. However, the critical issue is that the original care providers must continue to be involved [54].
- The time-out procedure is a simple check with a standardized questionnaire to ensure that the right operation is being performed. The Joint Commission in the USA included time-out procedures in operating rooms as accreditation criteria. Roos [55] stated that time-out procedures should be used to ensure that all checkpoints are summarized and checked with the entire team before surgery.
- Harten et al. [56] stated that there should be risk analysis, preventive measures, and evaluation processes to prevent risks, as well as controlling mechanisms, in safety management systems. In their study, the minimum requirements of a safety management system include: (i) pro-active periodic risk inventory, (ii) incident and complication reporting, (iii) systematic and structured approach for improvements, and (iv) strategic and operational activities.

In the examined safety management methodologies, it can be seen that there are some drawbacks to each methodology. This situation emphasizes the need for a hybrid model, in order to ensure patient safety. In this study, the focal point is prioritizing the errors to create improvement mechanisms for safety management. For that reason, the initial stage of the proposed model highly depends on the safety assessment calculations; thus, methodologies were investigated in terms of attribute selection procedures. After a detailed examination of the initial steps of each methodology, FMEA was chosen as a proper method.

FMEA was designed in 1960 by The National Aeronautics and Space Administration (NASA); later, Ford Motors adopted and promoted it in 1977 [57]. Today, it is used as a risk assessment tool in various fields [58]. Luo and Chang [59] used FMEA as a proper tool in their safety innovation model, in which they linked customer requirements and safety. FMEA has been proven to be one of the most important early preventative initiatives for companies and its popularity is still increasing; however, in the literature, there are several studies which have listed the shortcomings of risk priority number (*RPN*) calculations [60,61,62]. In the traditional approach, the values of severity (S), occurrence/frequency (O), and detectability (D), along with the weights (if they exist) for each failure mode, are given by experts, according to their importance level (with a rating scale between 1 and 10) [57]. Then, the determined levels and weights are simply multiplied to obtain the overall *RPN*, which is presented in Equation (1). After the calculations are completed, the final *RPN* scores are listed, in descending order, and a priority list is achieved. However, in real-life cases, this simple structure—which is one of the strongest sides of the model—becomes one of the main reasons for the shortcomings of the model.

(1) RPN=S×O×D

Liu et al. [62] pointed out the problems in setting the weights of risk factors. In the traditional approach, weights are directly multiplied by the determined *RPN* numbers. This situation causes duplication of the *RPN* elements and violation of the measurements. Huang et al. [63] stated that one of the major flaws of the traditional FMEA method is its sensitive formulation. To overcome these issues, multiplicative score functions are generally used [64]. Multiplicative functions are used to obtain a consistent result by using specified importance weights. Severity, occurrence/frequency, and detectability weights are defined as $W_{S},\;W_{O},$ and $W_{D}$, respectively. Therefore, the new *RPN* is calculated to eliminate the deficiency of the traditional approach of FMEA, as follows:

(2) $$RPN=S^{W_{S}}\times O^{W_{o}}\times D^{W_{D}}$$

Karasan et al. [65] used a weighted formula in their risk assessment methodology and created a model for safety and critical effect analysis. Although their model was based on the basic idea of FMEA, their new form of the weighted *RPN* calculations provided better results. In this study, unique scores for each medical error are calculated based on the weighted *RPN* formulation. The underlying reason is that the weight differences between the dimensions *S*, *O*, and *D* directly affect the final score result. Therefore, it is considered that it causes significant differences in real-life practice, especially in terms of prioritizing medical errors.

Healthcare systems are not deterministic, in terms of their structures. Analyzing the system is quite difficult, as the healthcare system itself and each of the factors affecting the system are probabilistic and uncertain [66,67]. It is almost impossible, in many cases, to form definitive definitions of real-life applications in the health field or to completely explain the relationships between medical concepts. Thus, the imperfect knowledge situation is inevitable and this structure of medical data causes uncertainty in the decision-making process [68,69]. For this reason, interval-valued intuitionistic fuzzy sets are considered as a proper methodology in this study, in terms of the structure of the health data.

## 3. Materials and Methods

### 3.1. Preliminaries

It is difficult to model systems, which are affected by people’s decisions, feelings, and perceptions using numerical values, especially in uncertain environments. For this reason, it is necessary to use linguistic expressions, which are words that substitute for exact numbers; that is, variables that are natural or artificial expressions. Each linguistic term is represented by a membership function, where a value of one represents that the related element is completely a member of the set.

A membership function for a fuzzy set A, which is defined as a subset of universal set E, is represented by $\mu_{A}\left(x\right):E\to \left[0,1\right]$. In this equation $\mu_{A}\left(x\right)$ represents the membership degree to set A. Thus, a fuzzy set A is defined by $A=\left\{\mu_{A}\left(x\right),\;x\right\}$. Over time, the scope of the theory has been expanded and used in the analysis of processes that have different data systems. In basic fuzzy sets, which are also called “Type-I Fuzzy Sets”, the membership function takes a certain value in the range [0,1]. On the other hand, this structure creates some basic problems in different data systems. The main reason for this is that people in real-life applications are hesitant to give their preferences in any way; in other words, they may remain ambiguous and even undecided about their preference level.

Intuitionistic fuzzy sets can be considered as an extended version of fuzzy set theory which better models imperfect knowledge [70]; they are also called “Type-II Fuzzy Sets”. Intuitionistic fuzzy sets are generally defined by the following equation:

(3) $$A=\langle \left(x,{\;\mu }_{A}\left(x\right),v_{A}\;\left(x\right)\right):x\;\in X\rangle$$

where, for each element x, $\mu_{A}\left(x\right):X\;\to \;\left[0,1\right]$ is a defined membership function and $v_{A}\left(x\right):X\;\to \;\left[0,1\right]$ is a defined non-membership function. These functions have the property of $0\;\leq {\;\mu }_{A}\left(x\right)+v_{A}\left(x\right)\leq 1$. In this case, hesitation, $\pi_{A}\left(x\right):X\;\to \;\left[0,1\right]$ is defined by the following equation:

(4) $$\pi_{A}\left(x\right)=1-{\;\mu }_{A}\left(x\right)-v_{A}\left(x\right)$$

In intuitionistic fuzzy set theory, each choice is represented as a single point within the set. On the other hand, the data may exist in a structure that cannot be expressed with a single point, in terms of uncertainty or complexity, and should be regarded as a range rather than a single point. For this reason, Atanassov and Gargov [71] proposed to expand intuitive fuzzy set theory and developed it as interval-valued intuitionistic fuzzy set (IVIF) theory, in which $\tilde{A}=\langle \left(x,{\;\mu }_{\tilde{A}}\left(x\right),\;v_{\tilde{A}}\;\left(x\right)\right):x\;\in X\rangle$; where $\mathrm{IV}\left[0,1\right]$ represents the closed subsets, $\mu_{\tilde{A}}\left(x\right):X\;\to \;\mathrm{IV}\left[0,1\right]$ is defined as a membership function, and $v_{\tilde{A}}\left(x\right):X\;\to \;\mathrm{IV}\left[0,1\right]$ is defined as a non-membership function.

In interval form, the membership function can be expressed as $\mu_{\tilde{A}}\left(x\right)=\left[\mu_{\tilde{A_{L}}}\left(x\right),{\;\mu }_{\tilde{A_{U}}}\left(x\right)\right]$ and the non-membership function can be expressed as $v_{\tilde{A}}\left(x\right)=\left[v_{\tilde{A_{L}}}\left(x\right),\;v_{\tilde{A_{U}}}\left(x\right)\right]$. In this case, the general function is $\tilde{A}=\langle \left(x,\;\left[\mu_{\tilde{A_{L}}}\left(x\right),{\;\mu }_{\tilde{A_{U}}}\left(x\right)\right],\;\left[v_{\tilde{A_{L}}}\left(x\right),\;v_{\tilde{A_{U}}}\left(x\right)\right]\right):x\in X\rangle$, which has the property $0\leq \mu_{\tilde{A_{U}}}\left(x\right)+v_{{\overset{ˇ}{A}}_{U}}\left(x\right)\leq 1$. The hesitation level is represented by $\pi_{\tilde{A}}\left(x\right)=\left[\pi_{\tilde{A_{L}}}\left(x\right),\;\pi_{\tilde{A_{U}}}\left(x\right)\right]$, where $\pi_{\tilde{A_{L}}}\left(x\right)=1-\mu_{\tilde{A_{U}}}\left(x\right)-v_{\tilde{A_{U}}}\left(x\right)$ and $\;\pi_{\tilde{A_{U}}}\left(x\right)=1-\mu_{\tilde{A_{L}}}\left(x\right)-v_{\tilde{A_{L}}}\left(x\right)$. Interval-valued intuitionistic fuzzy (IVIF) sets are generally denoted by $\left(\left[a,b\right]\;;\;\left[c,d\right]\right)$, for convenience. Let $\tilde{\alpha_{1}}=\left(\left[a_{1},b_{1}\right]\;;\;\left[c_{1},d_{1}\right]\right)$ and $\tilde{\alpha_{2}}=\left(\left[a_{2},b_{2}\right]\;;\;\left[c_{2},d_{2}\right]\right)$ be any two IVIF sets. Then, their operational laws can be defined as follows;

(5) $$\bar{\tilde{\alpha_{1}}}=\left(\left[c_{1},d_{1}\right]\;;\;\left[a_{1},b_{1}\right]\right)$$

(6) $$\tilde{\alpha_{1}}+\tilde{\alpha_{2}}=\left(\left[a_{1}+a_{2}-a_{1}\cdot a_{2}\;,b_{1}+b_{2}-b_{1}\cdot b_{2}\right]\;;\;\left[c_{1}\cdot c_{2\;},d_{1}\cdot d_{2}\right]\right)$$

(7) $$\tilde{\alpha_{1}}\cdot \tilde{\alpha_{2}}=\left(\left[a_{1}\cdot a_{2}\;,b_{1}\cdot b_{2}\right]\;;\;\left[c_{1}+c_{2\;}-c_{1}\cdot c_{2\;},d_{1}+d_{2}-d_{1}\cdot d_{2}\right]\right)$$

(8) $$\lambda \cdot \tilde{\alpha_{1}}=\left(\left[1-{\left(1-a_{1}\right)}^{\lambda }\;,\;1-{\left(1-b_{1}\right)}^{\lambda }\right]\;;\;\left[c_{1}^{\lambda }\;,d_{1}^{\lambda }\right]\right)$$

where λ>0.

Let $\tilde{\alpha_{1}}=\left(\left[0.1\;,\;0.2\right]\;;\;\left[0.5\;,\;0.5\right]\right)$ and $\tilde{\alpha_{2}}=\left(\left[0.2\;,\;0.3\right]\;;\;\left[0.5\;,\;0.6\right]\right)$ be two IVIFs. The complement of the first IVIF set is ${\tilde{\alpha_{1}}}^{C}=\left(\left[0.5\;,\;0.5\right]\;;\;\left[0.1\;,\;0.2\right]\right)$. Based on Equation (6), the summation of these IVIF sets is a new IVIF set equal to $\left(\left[0.28\;,\;0.44\right]\;;\;\left[0.25\;,\;0.3\right]\right)$, as $\tilde{\alpha_{1}}+\tilde{\alpha_{2}}=\left(\left[0.1+0.2-\left(0.1\times 0.2\right)\;,\;0.2+0.3-\left(0.2\;\times \;0.3\right)\right]\;;\;\left[\left(0.5\;\times \;0.5\right)\;,\;\left(0.5\;\times \;0.6\right)\right]\right).$ Similarly, the multiplication of sets gives a new IVIF set, as $\tilde{\alpha_{1}}\cdot \tilde{\alpha_{2}}=\left(\left[\left(0.1\;\times \;0.2\right),\left(0.2\;\times \;0.3\right)\right]\;;\;\left[0.5+0.5-\left(0.5\;\times \;0.5\right)\;,\;0.5+0.6-\left(0.5\;\times \;0.6\right)\right]\right)=\left(\left[0.02\;,\;0.06\right]\;;\;\left[0.75\;,\;0.8\right]\right)$, according to Equation (7). Suppose that λ=2. In this case, multiplication of the first IVIF set with λ gives $\left(\left[0.19\;,\;0.36\right]\;;\;\left[0.25\;,\;0.25\right]\right).$

Xu [72] proved the following results regarding the basic operations of IVIF sets:

(9) $$\tilde{\alpha_{1}}+\tilde{\alpha_{2}}=\tilde{\alpha_{2}}+\tilde{\alpha_{1}}\;$$

(10) $$\tilde{\alpha_{1}}\cdot \tilde{\alpha_{2}}=\tilde{\alpha_{2}}\cdot \tilde{\alpha_{1}}$$

(11) $$\lambda \cdot \left(\tilde{\alpha_{1}}+\tilde{\alpha_{2\;}}\right)=\lambda \cdot \tilde{\alpha_{1}}+\lambda \cdot \tilde{\alpha_{2}}$$

where λ≥0

(12) $$\lambda_{1}\cdot \tilde{\alpha_{1}}+\lambda_{2}\cdot \tilde{\alpha_{1}}=\left(\lambda_{1}+\lambda_{2}\right)\cdot \tilde{\alpha_{1}}\;$$

where $\lambda_{1},\;\lambda_{2}\;\geq 0$

Let us consider the same IVIF sets as an example, where $\tilde{\alpha_{1}}=\left(\left[0.1\;,\;0.2\right]\;;\left[0.5\;,\;0.5\right]\right)$ and $\tilde{\alpha_{2}}=\left(\left[0.2\;,\;0.3\right]\;;\left[0.5\;,\;0.6\right]\right)$. Equations (9) and (10) can be directly proven, since $\tilde{\alpha_{2}}+\tilde{\alpha_{1}}$ is also equal to $\left(\left[0.28\;,\;0.44\right]\;;\left[0.25\;,\;0.3\right]\right)$ and $\tilde{\alpha_{2}}\times \tilde{\alpha_{1}}$ is equal to $\left(\left[0.02\;,\;0.06\right]\;;\left[0.75\;,\;0.8\right]\right),$ according to Equations (6) and (7). Suppose that λ=2. Then, $\lambda \cdot \tilde{\alpha_{1}}=\left(\left[0.19\;,\;0.36\right]\;;\left[0.25\;,\;0.25\right]\right)$ and $\lambda \cdot \tilde{\alpha_{2}}=\left(\left[0.36\;,\;0.51\right]\;;\left[0.25\;,\;0.36\right]\right)$, based on Equation (8). In this case $\lambda \cdot \tilde{\alpha_{1}}+\lambda \cdot \tilde{\alpha_{2}}=\left(\left[0.19\;,\;0.36\right]\;;\left[0.25\;,\;0.25\right]\right)+\left(\left[0.36\;,\;0.51\right]\;;\left[0.25\;,\;0.36\right]\right)=\left(\left[0.4816\;,\;0.6864\right]\;;\left[0.0625\;,\;0.09\right]\right)$, which is also directly equal to $\lambda \cdot \left(\tilde{\alpha_{1}}+\tilde{\alpha_{2\;}}\right)=2.\left(\left[0.28\;,\;0.44\right]\;;\left[0.25\;,\;0.3\right]\right)$, as stated in Equation (11). Suppose that there are different λ values; for example, $\lambda_{1}=2\;$ and $\lambda_{2}=3$. Considering the first IVIF set $\lambda_{1}\cdot \tilde{\alpha_{1}}=\left(\left[0.19\;,\;0.36\right]\;;\left[0.25\;,\;0.25\right]\right)$ and $\lambda_{2}\cdot \tilde{\alpha_{1}}=\left(\left[0.271\;,\;0.488\right]\;;\left[0.125\;,\;0.125\right]\right)$; so, using Equation (6), the summation of these new IVIF sets can be found to give $\left(\left[0.4095\;,\;0.6723\right]\;;\left[0.03125\;,\;0.03125\right]\right),$ which is also found with $\left(\lambda_{1}+\lambda_{2}\right)\cdot \tilde{\alpha_{1}}=5.\;\left(\left[0.1\;,\;0.2\right]\;;\left[0.5\;,\;0.5\right]\right)=\left(\left[0.4095\;,\;0.6723\right]\;;\left[0.03125\;,\;0.03125\right]\right)$, as presented in Equation (12).

IVIFs can be aggregated with a weighted averaging operator [72]. This operator has been proven and used in many studies in the literature. Let $w=\left(w_{1},\;w_{2},\;\ldots ,\;w_{n}\right)$ be the weight vector for IVIF sets, where $\overset{n}{\underset{j=1}{\sum }}w_{j}=1$ and $w_{j}\;\in \;\left[0\;,1\right].$ The aggregation operator for IVIF sets is derived from an intuitionistic aggregation operation (IFWA) and is introduced as the IIFWA operator, which is calculated as follows [73];

(13) $$\mathrm{IIFWAw}\;\tilde{\alpha_{1}},\;\tilde{\alpha_{2}},\;\ldots ,\;\tilde{\alpha_{n}})=\left(\left[1-\overset{n}{\underset{j=1}{\prod }}{\left(1-\tilde{a_{j}}\right)}^{w_{j}}\;,\;1-\overset{n}{\underset{j=1}{\prod }}{\left(1-\tilde{b_{j}}\right)}^{w_{j}}\right];\left[\overset{n}{\underset{j=1}{\prod }}{\tilde{c_{j}}}^{w_{j}},\;\overset{n}{\underset{j=1}{\prod }}{\tilde{d_{j}}}^{w_{j}}\right]\right)$$

Suppose two IVIF sets $\tilde{\alpha_{1}}=\left(\left[0.1\;,\;0.2\right]\;;\;\left[0.5\;,\;0.5\right]\right)$ and $\tilde{\alpha_{2}}=\left(\left[0.2\;,\;0.3\right]\;;\;\left[0.5\;,\;0.6\right]\right)$ have weights $w_{1}=0.4$ and $w_{2}=0.6$, respectively. In this case, IIFWAw($\tilde{\alpha_{1}},\;\tilde{\alpha_{2}})=\left(\left[1-\overset{2}{\underset{j=1}{\prod }}{\left(1-\tilde{a_{j}}\right)}^{w_{j}}\;,\;1-\overset{2}{\underset{j=1}{\prod }}{\left(1-\tilde{b_{j}}\right)}^{w_{j}}\right]\;;\left[\overset{2}{\underset{j=1}{\prod }}{\tilde{c_{j}}}^{w_{j}},\;\overset{2}{\underset{j=1}{\prod }}{\tilde{d_{j}}}^{w_{j}}\right]\right)=\left(\left[1-\left(0.9^{0.4}\times 0.8^{0.6}\right),\;1-\left(0.8^{0.4}\times 0.7^{0.6}\right)\right]\;;\left[\left(0.5^{0.4}\times 0.5^{0.6}\right),\;\left(0.5^{0.4}\times 0.6^{0.6}\right)\right]\right)=\left(\left[0.426\;,\;0.262\right]\;;\;\left[0.500\;,\;0.558\right]\right)$.

In many multi-criteria decision-making problems, there are several decision-makers and their weights generally differ. In such cases, the IIFWA operator should be extended to consist of opinions of each of the decision-makers and all individual evaluations should be merged into one matrix. Let $R\;\left(mxn\right)$ represent the decision-makers evaluation matrix and $r_{ij}$ be a given element of this matrix. Suppose that the weight of the $k^{th}$ decision-maker is known and indicated by $\lambda_{k}$. When the IIFWA is adopted, the overall evaluation matrix (which consists of every decision-maker’s opinion) is calculated as follows.

(14) $$r_{ij}=\left(\left[1-\overset{n}{\underset{k=1}{\prod }}{\left(1-\tilde{a_{k}}\right)}^{\lambda_{k}}\;,\;1-\overset{n}{\underset{k=1}{\prod }}{\left(1-\tilde{b_{k}}\right)}^{\lambda_{k}}\right];\left[\overset{n}{\underset{k=1}{\prod }}{\tilde{c_{k}}}^{\lambda_{k}},\;\overset{n}{\underset{k=1}{\prod }}{\tilde{d_{k}}}^{\lambda_{k}}\right]\right)$$

where $\lambda_{k}\;\geq \;0,\;k\in 0,1,2\ldots n\;\mathrm{and}\;\overset{n}{\underset{k=1}{\sum }}\lambda_{k}=1$. Suppose that there are two decision-makers with different weights, such as $w_{1}=0.3$ and $w_{2}=0.7,$ respectively. Their evaluations for an alternative *i* according to the criteria *j* can be represented by IVIF sets, as $\tilde{\alpha_{1}}=\left(\left[0.1\;,\;0.2\right];\left[0.5\;,\;0.5\right]\right)$ and $\tilde{\alpha_{2}}=\left(\left[0.2\;,\;0.3\right];\left[0.5\;,\;0.6\right]\right)$. In accordance with Equation (14), the element $r_{ij}$ in the aggregated evaluation matrix is equal to $\left(\left[1-\overset{2}{\underset{k=1}{\prod }}{\left(1-\tilde{a_{k}}\right)}^{\lambda_{k}}\;,\;1-\overset{2}{\underset{k=1}{\prod }}{\left(1-\tilde{b_{k}}\right)}^{\lambda_{k}}\right];\left[\overset{2}{\underset{k=1}{\prod }}{\tilde{c_{k}}}^{\lambda_{k}},\;\overset{2}{\underset{k=1}{\prod }}{\tilde{d_{k}}}^{\lambda_{k}}\right]\right)=\left(\left[1-\left(0.9^{0.3}\times 0.8^{0.7}\right),\;1-\left(0.8^{0.3}\times 0.7^{0.7}\right)\right];\left[\left(0.5^{0.3}\times 0.5^{0.7}\right),\;\left(0.5^{0.3}\times 0.6^{0.7}\right)\right]\right)=\left[0.171\;,\;0.601\right];\left[0.500\;,\;0.568\right])$.

In comparing two IVIF sets, accuracy functions have been widely used [74,75,76,77]. In each study, to generate a better ranking system, several forms of accuracy functions are developed. Nyugen [78] stated that accuracy functions are a widely used method in decision-making problems, comparing and ranking the alternatives which are in the IVIF set structure. Garg [77] stated that studies conducted previously were unable to present the appropriate ranking between two IVIF sets. One study [77] suggested using a generalized structure for the score function, in which it is possible to evaluate the degree of hesitation between membership and non-membership functions. In other terms, parameters—namely, $k_{1}$ and $k_{2}$—are assigned to the membership and non-membership sets to represent the degree of hesitancy. The generalized improved score (GIS) function of an IVIF set can be calculated as follows:

(15) $$GIS\;\left(A\right)=\frac{a+b}{2}+k_{1}a\left(1-a-c\right)+k_{2}b\left(1-b-d\right),GIS\left(a\right)\in \left[0,1\right]$$

where $k_{1}+k_{2}=1\;\mathrm{and}\;k_{1},k_{2}\geq 0$. Increases in the parameter $k_{1}$ represents increased importance of the hesitation degree of the membership function, whereas an increase in the parameter $k_{2}$ represents the increased importance of the hesitation degree of the non-membership function. The change of values of the parameters represent the “attitudinal characters of the GIS” [77]. As an example, suppose $\tilde{\alpha_{1}}=\left(\left[0.1\;,\;0.2\right];\left[0.5\;,\;0.5\right]\right)$, $k_{1}=0.25,$ and $k_{2}=0.75$. Then, $GIS\left(\tilde{\alpha_{1}}\right)=\frac{0.1+0.2}{2}+\left(0.25\times 0.1\times \left(1-0.1-0.5\right)\right)+\left(0.75\times 0.2\times \left(1-0.2-0.5\right)\right)=0.205$. In this example, more importance is given to the hesitation degree of the non-membership function. On the other hand, if the parameters were changed, such as $k_{1}=0.75\;$ and $k_{2}=0.25$, then the GIS is also changed (for this example, it would be 0.195) and more importance is given to the hesitation degree of the membership function. In this study, the methodology proposed by Garg [77] is used for the final importance score calculations; however, the calculations are repeated by applying all mentioned methodologies.

Zhai et al. [79] indicated that there are basically three common kinds of measures—distance measures, similarity measures, and entropy measures—which are vital in solving decision-making problems to find the differences among elements. Especially for fuzzy sets, different forms of measures have been generated for IFs and IVIFs [79]. Similarity measurements are one of the most important and popular topics in fuzzy theory, as they can be used for a wide range of issues. Li et al. [34] stated that similarity measures between two sets should satisfy the following conditions: $S\left(A,\;B\right)$ is said to be a similarity measure between two sets *A* and *B* if:

(16) $$P1:\;S\left(A,\;B\right)\in \;\left[0,1\right]$$

(17) $$P2:\;S\left(A,\;B\right)=1\;⟺A=B\;\left(\mathrm{coincidence}\right)$$

(18) $$P3:\;S\left(A,\;B\right)=S\left(B,\;A\right)\;\left(\mathrm{symmetry}\right)$$

(19) $$P4:\;S\left(A,\;C\right)\leq S\left(A,\;B\right)\;and\;S\left(A,\;C\right)\leq S\left(B,\;C\right)\;if\;A\subseteq B\subseteq C\;\left(\mathrm{triangle}\;\mathrm{inequality}\right)$$

(20) $$P5:\;S\left(A,\;B\right)=0\;⟺A=\;\Phi \;and\;B=\bar{A}\;or\;A=\bar{B\;}\;and\;B=\Phi$$

In the literature, there are several forms of similarity calculations, as each researcher has tried to overcome the problems and irrational cases that were seen in previous methods. Xu and Chen [80] generalized the formulas of similarity measures for IVIFs. As was stated in the study [79], current definitions mostly focus on three-dimensional structures considering uncertainty.

Let $\tilde{A}$ and $\tilde{B}$ be two IVIF sets, where $\tilde{A}=\left\{\left(x_{j},\;\left[u_{A}^{-}\;\left(x_{j}\right),\;u_{A}^{+}\;\left(x_{j}\right)\right]\;,\;\left[v_{A}^{-}\;\left(x_{j}\right),\;v_{A}^{+}\;\left(x_{j}\right)\right]\right)\left|x_{j}\in X\right.\right\}$ and $\tilde{B}=\left\{\left(x_{j},\;\left[u_{B}^{-}\;\left(x_{j}\right),\;u_{B}^{+}\;\left(x_{j}\right)\right]\;,\;\left[v_{B}^{-}\;\left(x_{j}\right),\;v_{B}^{+}\;\left(x_{j}\right)\right]\right)\left|x_{j}\in X\right.\right\}.$ There are several similarity measures in the literature [33,81,82]. According to [81], the similarity between two IVIF sets *A* and *B* is calculated as follows:

$$M\left(\tilde{A},\tilde{B}\right)=\left\{\langle \left[u_{M\left(A,B\right)}^{-}\left(x\right),\;u_{M\left(A,B\right)}^{+}\left(x\right)\right],\;\left[v_{M\left(A,B\right)}^{-}\left(x\right),\;v_{M\left(A,B\right)}^{+}\left(x\right)\right]\rangle \left|x\;\in X\right.\right\}\;$$

where

$$u_{M\left(A,B\right)}^{-}\left(x\right)=min\left\{M_{AB1}\left(x\right),\;M_{AB2}\left(x\right)\right\}$$

$$u_{M\left(A,B\right)}^{+}\left(x\right)=max\left\{M_{AB1}\left(x\right),\;M_{AB2}\left(x\right)\right\}$$

$$v_{M\left(A,B\right)}^{-}\left(x\right)=min\left\{M_{AB3}\left(x\right),\;M_{AB4}\left(x\right)\right\}$$

(21) $$\;v_{M\left(A,B\right)}^{+}\left(x\right)=max\left\{M_{AB3}\left(x\right),\;M_{AB4}\left(x\right)\right\}$$

In this case

(22) $$M_{AB1}\left(x\right)=\frac{2+\left|u_{A}^{-}\left(x\right)-u_{B}^{-}\left(x\right)\right|+\left|v_{A}^{-}\left(x\right)-v_{B}^{-}\left(x\right)\right|-\left|\pi_{A}^{+}\left(x\right)+\pi_{B}^{+}\left(x\right)\right|}{4}$$

(23) $$M_{AB2}\left(x\right)=\frac{2+\left|u_{A}^{+}\left(x\right)-u_{B}^{+}\left(x\right)\right|+\left|v_{A}^{+}\left(x\right)-v_{B}^{+}\left(x\right)\right|-\left|\pi_{A}^{-}\left(x\right)+\pi_{B}^{-}\left(x\right)\right|}{4}$$

(24) $$M_{AB3}\left(x\right)=\frac{2-\left|u_{A}^{-}\left(x\right)-u_{B}^{-}\left(x\right)\right|+\left|v_{A}^{-}\left(x\right)-v_{B}^{-}\left(x\right)\right|-\left|\pi_{A}^{+}\left(x\right)+\pi_{B}^{+}\left(x\right)\right|}{4}$$

(25) $$M_{AB4}\left(x\right)=\frac{2-\left|u_{A}^{+}\left(x\right)-u_{B}^{+}\left(x\right)\right|+\left|v_{A}^{+}\left(x\right)-v_{B}^{+}\left(x\right)\right|-\left|\pi_{A}^{-}\left(x\right)+\pi_{B}^{-}\left(x\right)\right|}{4}$$

According to [81], the similarity between two IVIF sets A and B is $S\left(\tilde{A},\tilde{B}\right)=E\;\left(M\left(\tilde{A},\tilde{B}\right)\right)$, where “E” represents an IVIF entropy measure. Based on the given entropy calculations (Wu et al., 2014), it is possible to rearrange the similarity formula as follows:

$$S\left(\tilde{A},\tilde{B}\right)=\frac{1}{n}\overset{n}{\underset{j=1}{\sum }}\frac{4-\left(u_{j}^{-}+u_{j}^{+}+v_{j}^{-}+v_{j}^{+}\right)+\left(\pi_{j}^{-}+\pi_{j}^{+}\right)}{4+\left(u_{j}^{-}+u_{j}^{+}+v_{j}^{-}+v_{j}^{+}\right)+\left(\pi_{j}^{-}+\pi_{j}^{+}\right)}$$

where

$$u_{j}^{-}=\left|u_{A}^{-}\left(x_{j}\right)-u_{B}^{-}\left(x_{j}\right)\right|$$

$$u_{j}^{+}=\left|u_{A}^{+}\left(x_{j}\right)-u_{B}^{+}\left(x_{j}\right)\right|$$

$$v_{j}^{-}=\left|v_{A}^{-}\left(x_{j}\right)-v_{B}^{-}\left(x_{j}\right)\right|$$

$$v_{j}^{+}=\left|v_{A}^{+}\left(x_{j}\right)-v_{B}^{+}\left(x_{j}\right)\right|$$

$$\pi_{j}^{-}=\left|\pi_{A}^{-}\left(x_{j}\right)+\pi_{B}^{-}\left(x_{j}\right)\right|$$

(26) $$\pi_{j}^{+}=\left|\pi_{A}^{+}\left(x_{j}\right)+\pi_{B}^{+}\left(x_{j}\right)\right|$$

Let $\tilde{\alpha_{1}}=\left(\left[0.1\;,\;0.2\right]\;;\;\left[0.5\;,\;0.6\right]\right)$ and $\tilde{\alpha_{2}}=\left(\left[0.2\;,\;0.3\right]\;;\;\left[0.5\;,\;0.6\right]\right)$ be two IVIFs. In this case, based on Equation (22), $M_{AB1}\left(x\right)=0.25*\left(2+0.1-0.7\right)=0.35$ and, similarly, $M_{AB2}\left(x\right)$=0.45, $M_{AB3}\left(x\right)=0.3,$ and $M_{AB4}\left(x\right)=0.4$, according to Equations (22)–(24). These values are used to calculate $u_{M\left(A,B\right)}^{-}\left(x\right),\;u_{M\left(A,B\right)}^{\mp }\left(x\right),\;v_{M\left(A,B\right)}^{-}\left(x\right),\;v_{M\left(A,B\right)}^{+}\left(x\right)$, as presented in Equation (21), to reach the final value of $M\left(\tilde{A},\tilde{B}\right).$ For the given IVIF sets, $u_{M\left(A,B\right)}^{-}\left(x\right)=0.35,\;u_{M\left(A,B\right)}^{+}\left(x\right)=0.45,\;v_{M\left(A,B\right)}^{-}\left(x\right)=0.3,\;v_{M\left(A,B\right)}^{+}\left(x\right)=0.4$ and, finally, $\;M\left(\tilde{A},\tilde{B}\right)=\left(\left[0.35\;,\;0.45\right]\;;\;\left[0.3\;,\;0.4\right]\right)$. If the similarity is calculated based on the entropy, then the similarity level between the given IVIF sets is equal to 0.92 (as $\;E\left(M\left(\tilde{A},\tilde{B}\right)\right)=\frac{1.2}{1.3}=0.92$). However, instead of using the entropy, the similarity between given IVIF sets can be calculated directly, using Equation (26). In this case,$\;u_{j}^{-}=\left|u_{A}^{-}\left(x_{j}\right)-u_{B}^{-}\left(x_{j}\right)\right|=\left|0.1-0.2\right|=\left|-0.1\right|=0.1$ and, similarly, $u_{j}^{+}=0.1,\;v_{j}^{-}=0,\;\;v_{j}^{+}=0,\;\;\pi_{j}^{-}=0.3,\;\pi_{j}^{+}=0.7$. Therefore, the similarity between the given IVIF sets is, again, equal to 0.92 (as $\;S\left(\tilde{A},\tilde{B}\right)=\frac{4-\left(0.1+0.1+0+0\right)+\left(0.3+0.7\right)}{4+\left(0.1+0.1+0+0\right)+(0.3+0.7}=\frac{4.8}{5.2}=0.92$).

In this study, calculation of the similarity values is repeated by applying all mentioned methodologies [33,81,82] during the analysis of the collected data. However, the final calculations depend on the measurement system generated by Wu et al. [81]. The underlying reason for this is the structure of the methodology, in which hesitation is considered more properly than in other methodologies. Their study [81] considered the hesitation degree and the influence of the related values to handle the mentioned problems. Furthermore, the efficiency of the proposed methodology was proved by comparison with other methodologies.

### 3.2. Steps of the Proposed Model

Management of patient safety is required in a comprehensive model; therefore, we planned to generate an index system in which all medical errors are evaluated, in terms of severity, preventability, and occurrence/frequency criteria. Health institutions can be evaluated based on this index system, as the occurrence/frequency of errors is related to the evaluated health institution; thus, each error has a unique RPN score. After an RPN score for each error is determined for the evaluated health institution, these scores are used to create index values by using similarity measurements. It is possible to create an index value in the level of each error, each department, or each health institution. Moreover, these values can also be used to create a prioritization order for improvements in a health institution and provide a continuous development cycle.

**Figure 1:**
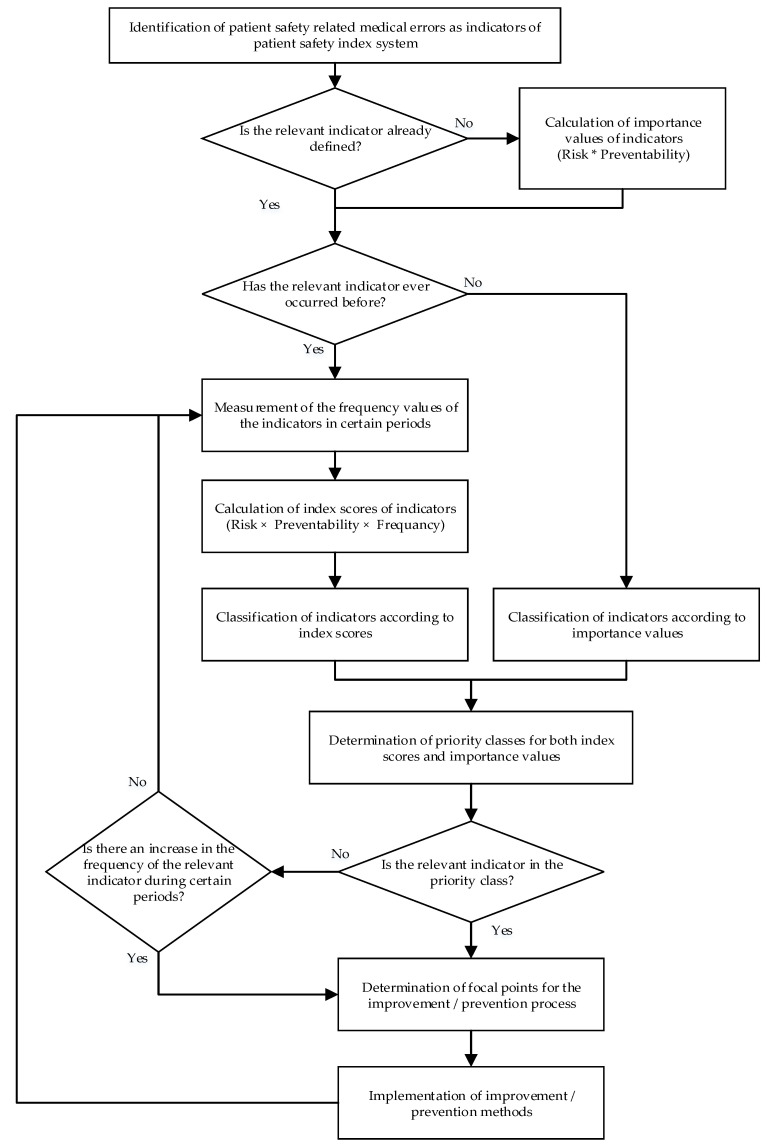
Proposed Patient Safety Index Methodology.

In the following section, each step of the proposed model (which is presented in Figure 1) is explained in detail. In this study, errors, which are most, reported in the safety reporting system of Turkey, are selected as examples to create a better understanding of the proposed model calculations.

#### 3.2.1. Step 1: Analyze the Actual Problem and Determine the Set of Attributes

It is not possible to completely eliminate the risk inherent to medical intervention, but it is necessary to weigh the risks before the decision stage. For this reason, after a review of the risk management literature and occupational health and safety regulations, attributes for the patient safety mechanism were selected as severity, preventability, and occurrence/frequency. Preventability represents how to prevent errors before they reach the patient or healthcare worker. Severity represents the impact of the damage that is caused, in terms of a related error in patients or health employees; in other terms, it is the degree of risk of the related error. Occurrence/frequency represents the number of occurred (reported) events for a specific error in an institution/health system.

In the determination of attribute weights, probabilistic linguistic term sets (for details, see [83]) and their basic formulations were used. An electronic evaluation form (Supplementary Materials), which was used to collect the opinions of decision-makers for importance grading, was applied for three months (August 2018–November 2018). A total of 138 answers were collected from all levels of health employees. According to the final calculation, the weights were determined as 0.387, 0.334, and 0.279 for severity, preventability, and occurrence/frequency, respectively.

After determination of the weights, attributes were evaluated for each error, in order to calculate an overall score based on a determined scaling. In this study, after a detailed literature review, scales for each attribute were determined as those presented in Table 2 and Table 3.

For severity and preventability evaluations, the linguistic terms presented in Table 2 and Table 3 were used to collect the opinions of decision-makers. These collected evaluations were used to calculate preventability and severity scores, according to corresponding IVIFs presented in Table 4. There are several types of IVIF scales in the literature; however, the underlying reason to prefer the scale given in Table 4 is because the scale should not consist of zero values.

For the determination of the occurrence/frequency scale, the risk management literature is investigated; however, risk assessment matrices are generally not suitable for healthcare systems. For that reason, the Palin perspective scale was adapted and used for the occurrence/frequency scale, as shown in Table 5.

For occurrence/frequency calculations, corresponding IVIFs for linguistic terms (which are presented in Table 6) were used. The main reason for using a different scale in the occurrence/frequency calculations was due to the structure of equations that were used for the prioritization calculations. In other terms, importance score calculations, which only consist of severity and preventability attributes were used to form the prioritization list; thus, the scale should not consist of zero values. However, in the index score calculations, performance of the related health institution/system is considered and similarity equations are used, which allow for the use of zero values. The literature was reviewed with this aim and one of the most-cited scales was chosen for the occurrence/frequency calculations, as presented in Table 6.

In the proposed model, the occurrence/frequency score of the related error was used directly after normalization of the number of patient values, since the occurrence/frequency score is unique for each health institution/health system.

#### 3.2.2. Step 2: Collection of Opinions of Decision-Makers

Decision-makers were asked to provide their opinions about each error, in terms of the attributes, severity, and preventability. First, the decision-makers were informed about the importance of patient safety; then, each attribute was explained, in detail, in terms of patient safety. When rating, the decision-makers evaluated the severity and preventability levels of the related error, based on their knowledge and experiences, using the determined linguistic IVIFs. Opinions of the decision-makers for error classification were collected using the electronic evaluation form presented in Supplementary Materials.

An electronic evaluation form was applied for a five-month period (June 2018–October 2018). The evaluation form was sent to all levels of health employees working in public/private hospitals in Turkey, especially doctors and nurses. Participants of the survey were generally from Istanbul, which has been ranked first, in terms of the number of hospitals and healthcare professionals. In total, 134 answers were collected; however, 12 of them eliminated because of the missing data. Finally, 122 answers were collected from all levels of health employees and evaluated in the study.

#### 3.2.3. Step 3: Aggregation of Opinions of Decision-Makers

Collected answers were in the form of linguistic scale codes. For that reason, all of the collected answers were first transformed into the scale version, using the given scales. After that, the IIFWA operator given in Equation (14) was used to aggregate the opinions of the decision-makers.

In Table 7 and Table 8, the aggregated values for the selected errors (most-occurring errors, based on the safety reporting system) are presented. In this study, decision-makers were thought to be at the same level; thus, they were assumed to have equal weights. However, in further studies, decision-makers can be weighted based on their experience and job title.

## 4. Results

In the proposed model, an RPN score is first calculated. Following this, these scores are used to find final index scores. Additionally, another unique value, called the importance score (in which only the severity and preventability of an error is considered), is calculated using accuracy calculations. The basic difference between index scores and importance scores is the occurrence/frequency value. If an error occurs in the related time period, the index scores are calculated; however, if an error is not reported, the importance score is calculated. The underlying reason for this is to take into consideration every error and to see the rankings of the errors regardless of the frequency.

### 4.1. Calculation of Index Scores

Index scores are calculated by using RPN scores, which include severity, preventability, and frequency/occurrence scores. In the determination of the frequency/occurrence scores, the number of reported events in 2016 was used. Then, the values were normalized. Finally, the RPN was found using Equation (2). The results are presented in Table 9.

Calculated RPNs were used to find overall index values. To determine the final prioritization similarity calculations, Equations (21)–(26) were used. In the calculations, the current state was compared with the ideal case. The ideal case refers to a “rare” evaluation for frequency/occurrence. Thus, an index value for each error was achieved as well, also presented in Table 9. High index scores mean that the related error is close to the ideal case; in other terms, these errors were rarely seen. For this reason, errors are listed in increasing order, based on their index values.

According to the calculated index scores, it was possible to create a prioritization for the related error. In Table 10, values for each error are presented, along with their ranking based on the number of reported issues.

In the current situation, the reported number of events is the only dimension that is considered when prioritizing the errors. If the reported number of errors was high, then the related error gains much more priority over the others and improvements should be planned based on this ranking. However, as mentioned before, it is not suitable to evaluate risks based only on frequency, especially for healthcare institutions. When the severity and the preventability of the errors were evaluated, the ranking completely changed, as shown in Table 10.

### 4.2. Calculation of Importance Scores

In the proposed model, if the error occurred in the observation period, index scores were accepted to create the ranking. However, an importance value for each error was also calculated, in order to determine the ranking of the errors regardless of their frequency. The main aim was taking precautions for errors in a systematic way; in other terms, the fact that an error has not occurred before does not mean that related error will not occur later. For this reason, the system should consider every dimension and preventive mechanisms should be taken into account, if it is deemed necessary. Thus, it is possible to evaluate the general position of health institutions from a proactive point of view.

Moreover, the calculation of an importance score is a precaution of the unreliable structure of the reporting system. In the given example, by examination of the report published by the Ministry of Health, Turkey [26], 93.82% (69.782) of reported events constituted laboratory-related errors. The underlying reason for this conclusion is directly related to the reporting culture. In other words, error reporting and follow-up structures are considered as a basic part of laboratory systems, which explains the encountered results. For that reason, errors were considered as if they have not occurred before, the importance value for each of them was calculated, and a different ranking was also established.

The weights of attributes were used to calculate a new RPN for each error, based on Equation (2). In the new RPN, the aggregated opinions of decision-makers only for severity and preventability (which were determined in Step 2) were used. Initially, these calculated scores were used for ranking the errors without considering the frequency/occurrence. This analysis refers to the “classification of errors based on the importance values” in Figure 1 and is presented in Table 11. In the ranking, the results are calculated by the accuracy function [77] given by Equation (15). In the accuracy calculations, different values of k_1_ and k_2_ are considered, as they change the importance given to the membership and non-membership hesitation degrees. It can be seen, from the table, that the basic ranking (final column) remained the same. This situation proves that the model is good at dealing with hesitation.

It can be seen that, if the reporting mechanism is good and the number of reported events reflects the actual number of errors occurring in health institutions, the index scores that are generated by the proposed model create a proper basis for improvement. On the other hand, even if the reporting mechanism is not organized very well, the important values that are generated by the proposed model still create a proper input.

## 5. Discussion

In this study, a new patient safety management model is presented, in which errors are evaluated based on their severity, preventability, and occurrence/frequency scores. In order to indicate the correct scores, the main advantages of the system are having a reliable error reporting mechanism and providing a basis for patient safety culture in health institutions. The proposed model provides valuable information and changes the ranking of errors, as shown in the analysis sections of the study. However, it is not possible to reach logical results without correct information in terms of frequency. For that reason, importance weight calculations were also added to the analyses, in order to overcome to this problem.

Simulation models can be used to increase the efficiency of reporting mechanisms. In the literature, there exist many studies which have tried to modify reporting systems, considering several scenarios [87]. At present, many of these efforts have focused only on medication-related errors, as the creation of simulation scenarios for them are easier than for other type of errors. Therefore, we plan to conduct a literature review on simulation modelling in patient safety as a further study. Then, we plan to develop a simulation model to achieve a better reporting mechanism, in which the proposed model is used for the overall calculations.

In addition to the prioritization of medical errors, a control mechanism was also added to the model, in which a trend is controlled; in other words, after errors are prioritized, the trend of the errors is also controlled (whether there is a constant increase or not). The main aim of this was to observe and take precautions with respect to those errors whose occurrence increases over time but are not classified in the prioritized class. These errors can be considered as hidden improvement points, but are generally ignored as they typically do not cause serious harm. However, they are important to consider when conducting an overall safety review of a health institution.

In this study, the evaluation of each error, in terms of severity and preventability, created a limitation, as the final determined list of the medical errors included over 200 errors, thus making collecting and aggregating the opinions of decision-makers a difficult issue. Another limitation is that each error can cause different outcomes in different patients. However, the proposed model provides a prioritization based on the general evaluation of the errors; that is, independently of the patient. Therefore, outcomes can be clustered and core reasons can be revealed, such that the occurrence of the same error can be prevented in a systematic way.

## 6. Conclusions

Modern healthcare institutions are generally defined as service organizations operating for the production and delivery of health services by coordinating all factors in a conscious, systematic, and harmonized way, in order to meet the health needs of people [88,89]. For that reason, healthcare providers have to respond to a growing demand for a good and acceptable quality of care. Recently, the implementation of several methodologies and the development of hybrid models have not only increased awareness but also generated an impulse for multidisciplinary perspectives in healthcare, in terms of patient safety.

In the future, as the healthcare system reforms, patient safety should remain a top priority. Day by day, more organizations are adopting new systems to reach high quality levels, achieving improvements in medication safety and trying to improve the patient safety culture. In this study, a comprehensive decision-making model is proposed, in order to better evaluate the current situation of health institutions and to create a basis for improvement stages. The index scores generated by the proposed model can be used to manage safety

The modern healthcare environment is continuously changing. It is estimated that global healthcare spending are projected to reach $8.7 trillion by 2020 and spending rates, for the world’s major regions, will increase from 2.4% to 7.5% [90]. However, in 2019, Deloitte [91] refreshed their estimations of healthcare spending, stating that spending will reach $10.059 trillion by 2022. Patient safety issues are important, in terms of the need for increased efforts to limit health costs and care management. In this framework, in the near future, healthcare will be put under pressure to offer a high level of quality, safety, and customer service continuously, while simultaneously ensuring improved efficiency.

## Figures and Tables

**Table 1:** Summary of the examined methodologies.

Methodology	Advantages	Disadvantages
Move your Dot	Detailed analysis (miscommunication, scheduling errors, adverse events, and non-response to a question are also evaluated).	Filling records is not a controlled process and requires improvements. Many problems can be encountered during the data collection phase.
	Tracking errors in the system.	
	Inter-hospital variation can be tracked.	
Trigger tool	Suitable for monitoring the number of adverse events (at the department level or a specific care level).	Screening and assessment take time, as assessment should ideally be made by two individuals independently and agreed upon.
Bow-tie model	Useful for analyzing the current safety management model.	Creating a complete model for each risk takes too much time and a number of sessions in which multidisciplinary focus is needed. Moreover, the success of the analysis highly depends on the motivation of the participants. Thus, it is too easy to set the wrong priorities.
	Easy determination of root causes.	
Health failure mode and effect analysis—(H)FMEA	Visible risks.	(H)FMEA takes too much time, as a minimum of four participants is needed. For this reason, the model is time-intensive. Time, resources, and space must be made available to implement the method properly.
	Helps to take precautions before an incident takes place.	
Root cause analysis (RCA)	Objective description	RCA is a time-consuming approach and not every employee is suitable to carry out the analysis. Moreover, the contributions of RCA to improving patient safety have not been scientifically presented.
	Create recommendations to prevent the occurrence of similar errors.	
	Creates insight into the employees.	
PRISMA	Detailed analysis with quantitative and qualitative insights.	In the model, after a casual tree and the classifications of the basic causes are made, a link with context variables for each basic cause should be created. If PRISMA is planned for use in comparing data between organizations, it is vital to design variables uniformly.
	Visible conclusions.	

**Table 2:** Preventability Scale (Adapted from [65]).

Code	Definition
1	Easily Preventable (control charts etc.)
2	Can be preventable with small system improvements
3	Can be preventable with training
4	Can be preventable with radical improvements
5	Unpreventable but side effects can be preventable
6	Unpreventable

**Table 3:** Severity Scale (Adapted from [84]).

Code	Definition
LI	Grade 0: Without repercussion for the patient (near miss)
MLI	Grade 1: Adverse events contributed to temporary harm to the patient and intervention was needed
MI	Grade 2: Contributed to temporary harm to the patient, thereby resulting in longer admission being necessary
I	Grade 3: Contributed to or resulted in permanent harm to the patient
VI	Grade 4: Intervention was needed to keep the patient alive
EI	Grade 5: Contributed to the death of the patient

**Table 4:** Linguistic Terms for Preventability and Severity with Corresponding Interval-Valued Intuitionistic Fuzzy Sets (IVIFs) (Adapted from [73]).

Linguistic Term (Code)		Membership Values (μ)	Non-Membership Values (v)
Preventability	Severity	$\left(\mu_{L}\;;\;\mu_{U}\right)$	$\;\left(v_{L}\;;\;v_{U}\right)$
1	EI	(0.65; 0.75)	(0.1; 0.25)
2	VI	(0.60; 0.70)	(0.15; 0.30)
3	I	(0.55; 0.65)	(0.20; 0.35)
4	MI	(0.50; 0.60)	(0.25; 0.40)
5	MLI	(0.45; 0.55)	(0.30; 0.45)
6	LI	(0.25; 0.40)	(0.50; 0.60)

**Table 5:** Occurrence/Frequency Scale (Adapted from [85]).

Code	Definition
Rare	Less than 1 in 100,000,000 (probability of being x < 0.00000001)
Very Low	1 in 100,000,000 (probability of being 0.00000001 < x < 0.0000001)
Low	1 in 10,000,000 (probability of being 0.0000001 < x < 0.000001)
Medium-Low	1 in 1,000,000 (probability of being 0.000001 < x < 0.0001)
Medium	1 in 100,000 (probability of being 0.00001 < x < 0.0001)
Medium High	1 in 10,000 (probability of being 0.0001 < x < 0.001)
High	1 in 1,000 (probability of being 0.001 < x < 0.01)
Very High	1 in 100 (probability of being 0.01 < x < 0.1)
Extremely High	1 in 10 (probability of being 0.1 < x < 1)

**Table 6:** Linguistic Terms for Occurrence/Frequency with Corresponding IVIFs (Adapted from [86]).

Linguistic Term for Occurrence/Frequency	Membership Values (μ)	Non−Membership Values (v)
	$\left(\mu_{L}\;;\;\mu_{U}\right)$	$\left(v_{L}\;;\;v_{U}\right)$
Rare	(0; 0.2)	(0.5; 0.8)
Very Low	(0.1; 0.3)	(0.4; 0.7)
Low	(0.2; 0.4)	(0.3; 0.6)
Medium-Low	(0.3; 0.5)	(0.2; 0.5)
Medium	(0.4; 0.6)	(0.2; 0.4)
Medium High	(0.5; 0.7)	(0.1; 0.3)
High	(0.6; 0.8)	(0; 0.2)
Very High	(0.7; 0.9)	(0; 0.1)
Extremely High	(0.8; 1)	(0; 0)

**Table 7:** Final Aggregated Scores for Errors, in Terms of Severity Evaluations.

Error Type	Code	Error Name	Membership Values (μ)	Non−Membership Values (v)
			$\left(\mu_{L}\;;\;\mu_{U}\right)$	$\left(v_{L}\;;\;v_{U}\right)$
Laboratory	L18	Clotted sample	(0.472; 0.572)	(0.277; 0.428)
	L17	Sample with hemolysis	(0.485; 0.585)	(0.264; 0.415)
	L16	Insufficient sample	(0.466; 0.570)	(0.281; 0.430)
	L05	Wrong record	(0.432; 0.550)	(0.303; 0.450)
	L01	Wrong test request	(0.415; 0.530)	(0.328; 0.470)
Medication	I1g	Erroneous dosing	(0.537; 0.637)	(0.211; 0.363)
	I1a	Wrong drug demand	(0.505; 0.605)	(0.244; 0.395)
	I0a	Inappropriate temperature and humidity	(0.410; 0.528)	(0.331; 0.472)
	I2b	Preparation of wrong drug	(0.538; 0.643)	(0.202; 0.357)
	I3c	Transferring wrong drug from pharmacy	(0.486; 0.590)	(0.259; 0.410)
General/Surgical	di	Non-marking of operation area/side	(0.551; 0.651)	(0.197; 0.349)
	kd	Unverified Patient ID, location of operation and surgical procedure	(0.529; 0.630)	(0.219; 0.370)
	gd	Not confirming removal of make-up, prosthesis and valuable items	(0.483; 0.585)	(0.264; 0.415)
	tr	The operation area has not been shaved	(0.491; 0.595)	(0.254; 0.406)
	ts	Health worker does not accompany patient transfer	(0.482; 0.582)	(0.267; 0.418)
Patient Related	HRHc	Patient Falls	(0.596; 0.697)	(0.146; 0.303)
	TKHg	Incorrect report of patient basic information	(0.531; 0.631)	(0.217; 0.369)
	TKHa	Incorrect identification of patients	(0.582; 0.684)	(0.157; 0.316)

**Table 8:** Final Aggregated Scores for Errors in Terms of Preventability Evaluations.

Error Type	Code	Error Name	Membership Values (μ)	Non−Membership Values (v)
			$\left(\mu_{L}\;;\;\mu_{U}\right)$	$\left(v_{L}\;;\;v_{U}\right)$
Laboratory	L18	Clotted sample	(0.553; 0.654)	(0.193; 0.346)
	L17	Sample with hemolysis	(0.555; 0.656)	(0.190; 0.344)
	L16	Insufficient sample	(0.586; 0.687)	(0.160; 0.313)
	L05	Wrong record	(0.559; 0.660)	(0.188; 0.340)
	L01	Wrong test request	(0.618; 0.718)	(0.130; 0.282)
Medication	I1g	Erroneous dosing	(0.511; 0.619)	(0.227; 0.382)
	I1a	Wrong drug demand	(0.533; 0.636)	(0.212; 0.364)
	I0a	Inappropriate temperature and humidity	(0.560; 0.700)	(0.147; 0.300)
	I2b	Preparation of wrong drug	(0.527; 0.631)	(0.217; 0.369)
	I3c	Transferring wrong drug from pharmacy	(0.566; 0.667)	(0.181; 0.333)
General/Surgical	di	Non-marking of operation area/side	(0.554; 0.658)	(0.183; 0.342)
	kd	Unverified Patient ID, location of operation and surgical procedure	(0.558; 0.663)	(0.180; 0.337)
	gd	Not confirming removal of make-up, prosthesis and valuable items	(0.576; 0.682)	(0.160; 0.318)
	tr	The operation area has not been shaved	(0.591; 0.696)	(0.146; 0.304)
	ts	Health worker does not accompany patient transfer	(0.572; 0.678)	(0.166; 0.322)
Patient Related	HRHc	Patient Falls	(0.543; 0.645)	(0.199; 0.356)
	TKHg	Incorrect report of patient basic information	(0.566; 0.671)	(0.175; 0.329)
	TKHa	Incorrect identification of patients	(0.547; 0.656)	(0.186; 0.344)

**Table 9:** Ranking of Errors Based on Index Scores (Preventability × Severity × Occurrence).

	RPN		Ideal Case		Similarity	
Code	$\left(\mu_{L}\;;\;\mu_{U}\right)$	$\left(v_{L}\;;\;v_{U}\right)$	$\left(\mu_{L}\;;\;\mu_{U}\right)$	$\left(v_{L}\;;\;v_{U}\right)$	Score	Ranking
L18	(0.532; 0.657)	(0.179; 0.343)	(0; 0.446)	(0.323; 0.554)	0.638	2
L17	(0.538; 0.663)	(0.172; 0.337)	(0; 0.451)	(0.318; 0.549)	0.635	1
L16	(0.513; 0.642)	(0.193; 0.358)	(0; 0.453)	(0.315; 0.547)	0.662	5
L05	(0.516; 0.649)	(0.189; 0.351)	(0; 0.441)	(0.331; 0.559)	0.644	4
L01	(0.525; 0.658)	(0.181; 0.342)	(0; 0.447)	(0.325; 0.553)	0.640	3
I1g	(0.486; 0.620)	(0.213; 0.380)	(0; 0.457)	(0.310; 0.543)	0.691	11
I1a	(0.482; 0.614)	(0.221; 0.386)	(0; 0.452)	(0.317; 0.548)	0.693	14
I0a	(0.462; 0.601)	(0.237; 0.399)	(0; 0.443)	(0.331; 0.557)	0.701	15
I2b	(0.492; 0.627)	(0.206; 0.373)	(0; 0.461)	(0.304; 0.539)	0.689	10
I3c	(0.484; 0.618)	(0.217; 0.382)	(0; 0.455)	(0.313; 0.545)	0.692	13
di	(0.505; 0.639)	(0.193; 0.361)	(0; 0.470)	(0.292; 0.530)	0.684	8
kd	(0.498; 0.632)	(0.201; 0.368)	(0; 0.465)	(0.299; 0.535)	0.686	9
gd	(0.486; 0.620)	(0.213; 0.380)	(0; 0.456)	(0.310; 0.544)	0.692	12
tr	(0.455; 0.597)	(0.204; 0.403)	(0; 0.462)	(0.302; 0.538)	0.719	17
ts	(0.447; 0.587)	(0.216; 0.413)	(0; 0.455)	(0.312; 0.545)	0.723	18
HRHc	(0.517; 0.651)	(0.179; 0.349)	(0; 0.479)	(0.280; 0.521)	0.679	6
TKHg	(0.462; 0.604)	(0.198; 0.396)	(0; 0.468)	(0.297; 0.532)	0.716	16
TKHa	(0.513; 0.650)	(0.179; 0.350)	(0; 0.479)	(0.280; 0.521)	0.680	7

**Table 10:** Comparison of Rankings.

Code	Ranking Based on Index Score	Ranking Based on the Reported Number of Events
L18	2	1
L17	1	2
L16	5	5
L05	4	4
L01	3	3
I1g	11	7
I1a	14	9
I0a	15	12
I2b	10	10
I3c	13	14
di	8	6
kd	9	11
gd	12	13
tr	17	17
ts	18	18
HRHc	6	8
TKHg	16	16
TKHa	7	15

**Table 11:** Ranking of Errors Based on the Importance of Values (Preventability × Severity).

Degree of Hesitancy	k_1_ = 0		k_1_ = 0.25		k_1_ = 0.50		k_1_ = 0.75		k_1_ = 1	
	k_2_ = 1		k_2_ = 0.75		k_2_ = 0.50		k_2_ = 0.25		k_2_ = 0	
Code	Score	Ranking	Score	Ranking	Score	Ranking	Score	Ranking	Score	Ranking
L18	0.559	15	0.591	15	0.623	15	0.655	15	0.687	15
L17	0.567	14	0.600	14	0.632	14	0.665	14	0.698	14
L16	0.570	12	0.603	12	0.636	12	0.669	12	0.702	12
L05	0.543	18	0.575	18	0.607	18	0.639	18	0.670	18
L01	0.555	16	0.587	16	0.620	16	0.652	16	0.684	16
I1g	0.576	9	0.610	9	0.644	9	0.678	9	0.711	9
I1a	0.569	13	0.601	13	0.634	13	0.667	13	0.700	13
I0a	0.546	17	0.578	17	0.610	17	0.641	17	0.673	17
I2b	0.585	7	0.619	7	0.654	7	0.688	7	0.723	7
I3c	0.573	11	0.606	11	0.640	11	0.673	11	0.706	11
di	0.603	3	0.639	3	0.674	3	0.710	3	0.745	3
kd	0.594	5	0.628	5	0.663	5	0.698	5	0.733	5
gd	0.576	8	0.610	8	0.644	8	0.678	8	0.712	8
tr	0.588	6	0.622	6	0.657	6	0.692	6	0.726	6
ts	0.573	10	0.607	10	0.640	10	0.674	10	0.707	10
HRHc	0.621	1	0.658	1	0.695	1	0.732	1	0.769	1
TKHg	0.598	4	0.633	4	0.668	4	0.703	4	0.738	4
TKHa	0.618	2	0.655	2	0.693	2	0.730	2	0.767	2

## Supplementary Materials

The following are available online at https://www.mdpi.com/2227-9032/8/3/265/s1, Evaluation forms.
