# Supplementary material for: Prioritization of Medical Errors in Patient Safety Management: Framework Using Interval-Valued Intuitionistic Fuzzy Sets
Source: Healthcare (Basel). 2020 Aug 12;8(3):265. doi: 10.3390/healthcare8030265 (PMC7551010; doi:10.3390/healthcare8030265)
Supplement: Supplementary file 1 [file healthcare-08-00265-s001.pdf]

healthcare

MDPI

Evaluation Forms

Importance Grading Evaluation Form

IMPORTANCE GRADING EVALUATION

Dear Participant,

Patient safety means preventing the mistakes in the health processes from causing unnecessary harm to the patient and the healthcare worker, or avoiding the potential harm, and includes the measures taken to prevent the harm of health services to individuals.

In order to ensure that errors are identified, reported, and corrected before reaching patients and healthcare professionals, the characteristics of the errors must be evaluated. In this study, the relevant errors are examined in terms of risk, preventability level, and frequency of incidence.

We need the opinions of you, the valuable participants, in order to obtain a correct and meaningful result with the planned work.

- **Severity:** It represents the effect of the harm that the related error will have on the patient or healthcare worker. For this category, you are asked to state your opinion on how important the impact level is in the evaluation of errors.
- **Preventability:** Represents how the relevant error can be prevented before it reaches the patient or healthcare professional. For this category, you are asked to indicate your opinion about how important the level of preventability is in the evaluation of errors.
- **Frequency/Occurrence:** Represents the number of times the relevant error is encountered in an institution (the number of patients or healthcare professionals it affects). For this category, you are asked to indicate your opinion about how important the level of incidence in the evaluation of errors.

Thank you for expressing your views using the criteria presented…

**Question 1: Please evaluate the importance of the mentioned criteria over 10 for any type of error that may occur in terms of patient safety. (Total of your evaluations should be 10)**
Severity: ___<br>Preventability: ___<br>Frequency: ___
**If you have any other opinion than your assessment for Question 1, please fill in the second evaluation section below. Otherwise, go to the next question by sliding the page.**
Please evaluate the importance of the mentioned criteria over 10 for any type of error that may occur in terms of patient safety. (Total of your evaluations should be 10)<br>Severity: ___<br>Preventability: ___<br>Frequency: ___
**If you have any other opinion than the two different evaluations, you made for Question 1, please fill in the third evaluation section below. Otherwise, go to the next question by sliding the page.**

Please evaluate the importance of the mentioned criteria over 10 for any type of error that may occur in terms of patient safety. (Total of your evaluations should be 10)

Severity:

Preventability:

Frequency:

Question 2. Please rate your suggestions out of 100. (Total of your evaluations must be 100) Your answer to this question represents the strength of your opinion on the ratings you have specified.

1. Rating:

2. Rating:

3. Rating:

Please indicate if you have any additional comments on the rating recommendations stated. Otherwise, you can leave the blank and complete the survey.

Error Classification System Evaluation Form

Dear Participant,

Patient safety means preventing the mistakes in the health processes from causing unnecessary harm to the patient and the healthcare worker, or avoiding the potential harm, and includes the measures taken to prevent the harm of health services to individuals.

In order to ensure that errors are identified, reported, and corrected before reaching patients and healthcare professionals, the characteristics of the errors must be evaluated. In this study, the relevant errors are examined in terms of severity and preventability level.

We need the opinions of valuable participants, in order to obtain a correct and meaningful result with the planned work.

- **Severity:** It represents the effect of the harm that the related error will have on the patient or healthcare worker. For this category, you are asked to state your opinion about the severity level of each error via using following scale.
- **Preventability:** Represents how the relevant error can be prevented before it reaches the patient or healthcare professional. For this category, you are asked to indicate your opinion about each errors' level of preventability via using following scale.

Severity Level		*Preventability Level*	
Coding	**Definition**	***Coding***	**Definition**
EI	Contributed to the death of the patient.	***1***	Easily preventable (control charts etc.)

VI	Intervention was needed to keep the patient alive.	***2***	Can be preventable with small system improvements.
I	Contributed to or resulted in permanent harm to the patient.	***3***	Can be preventable with trainings.
MI	Contributed to the temporary harm to the patient, thereby resulting in longer admission.	***4***	Can be preventable with radical improvements.
MLI	Adverse events contributed to temporary harm to the patient and intervention was needed.	***5***	Unpreventable but side effects can be preventable.
LI	Without repercussion for the patient (near miss).	***6***	Unpreventable.

Thank you for your valuable contribution

*** LABORATORY ERRORS**

**Pre-Analytic, Analytic and Post-Analytic Sub-Errors

	Severity Level	Preventability Level
Clotted sample		
Sample with hemolysis		
Insufficient sample		
Wrong record		
Wrong test request		
……		

*** MEDICATION ERRORS**

**Storage, Demand, Preparation, Transfer, Implementation and Post-implementation Sub-Errors

	Severity Level	Preventability Level
Erroneous dosing		
Wrong drug demand		
Inappropriate temperature and humidity		
Preparation of the wrong drug		
Transferring the wrong drug from the pharmacy		
….		

*** GENERAL/SURGICAL ERRORS**

**Clinical Surgical Procedure Preparation, Transfer to the Operating Room and Patient Acceptance, Operating Room Surgical Procedure, Pre-Anesthesia Preparation and Control, Controls Before Surgery, Follow-up and Control During Operation, Controls at the End of the Operation, Departure of the Patient from the Operation Room and Transfer Sub-Errors

	Severity Level	Preventability Level
Non-marking of operation area/side		
Unverified patient ID, location of operation and surgical procedure		
Not confirming removal of make-up, prosthesis and valuable items		
The operation area has not been shaved		
Health worker does not accompany patient transfer		
…..		

*** PATIENT RELATED ERRORS**

**Medical Record and Clinic Evaluation Related, Care, Diagnosis and Treatment Process Related, Errors Concerning the Transfusion of Blood and Blood Products, Errors Regarding Nutrition Patient/Attendant Related and Communication Errors, Physical Structure Related or Device/Equipment/System Related Errors

	Severity Level	Preventability Level
Patient Falls		
Incorrect report of patient basic information		

Incorrect identification of patients		
….		

* Main category of the medical errors. ** Sub-categories of the related main category.
